# Determinants and prevalence of metabolic syndrome among the adult population in Hargeisa, Somaliland: A community-based cross-sectional study

**DOI:** 10.1371/journal.pone.0316094

**Published:** 2024-12-27

**Authors:** Abdeta Muktar Ahmed, Ayanle Suleiman Ahmed, Mohamed Mussa Abdilahi, Abdulkadir Mohamed Nuh

**Affiliations:** 1 College of Medicine and Health Sciences, University of Hargeisa, Hargeisa, Somaliland; 2 Department of Nutrition, Addis Ababa Medical University College Hargeisa Campus, Hargeisa, Somaliland; Hamadan University of Medical Sciences, School of Public Health, ISLAMIC REPUBLIC OF IRAN

## Abstract

**Background:**

Metabolic syndrome (met-s) is a medical condition that includes abdominal obesity, hyperlipidemia, high blood glucose, and high blood pressure. It is associated with a high risk of developing cardiovascular diseases and type 2 diabetes mellitus. The condition was believed to be a challenge mostly faced by developed nations. A few studies conducted showed that met-s is increasing and becoming more common in Africa, where it was considered rare. The study aimed to assess the determinants and prevalence of met-s among the adult population in Hargeisa town, Somaliland, in 2023.

**Methods:**

A community-based cross-sectional study among 498 adults living in all eight districts of Hargeisa, was carried out from August to September 2023. The sample size was divided proportionally by the number of households in selected sub-districts. Systematic random sampling was employed to select the households in the sub-districts. One adult from each household was selected and assessed. Data were collected using the STEPwise approach of the World Health Organization. The data were analysed using International Diabetic Federation (IDF) criteria for metabolic syndrome with SPSS version 25. Bivariate and multivariate analyses using logistic regression were performed.

**Result:**

In total, 498 adults participated in the study. The prevalence of met-s was 26.7% in IDF (males 11% vs. females 38.9%). Being of an advanced age of 45–54 years (AOR = 3.6, CI 1.17–11.27), 55–64 years (AOR = 6.1, CI 1.88–19.83), >64 (AOR = 9.1 CI 2.41–34.92), being a woman (AOR = 10.8, CI 5.3–21.8), being overweight or obese (AOR = 4.5, CI 2.5–8), sedentary behavior (AOR = 3.5, CI 1.6–7.5), and lack of physical exercise (AOR = 0.39, CI 0.17–0.88) were significantly associated with met-s.

**Conclusion:**

The met-s was predominant in our findings. Community-based prevention strategies and actions are necessary if the met-s and its potential consequences are needed to be mitigated.

## Background

According to WHO (World Health Organization) report, every year non-communicable diseases (NCD) cause 17 million deaths around the globe before the age of 70 years, in which low- and middle-income nations account for 86% of these premature deaths [1]. The metabolic syndrome (met-s) which is characterized by abdominal obesity, hyperlipidemia, elevated blood glucose, and elevated blood pressure, is one of the major global public health challenges, and is associated with a high risk of developing NCDs such as cardiovascular diseases (CVD) and type 2 diabetes mellitus. Visceral fat and insulin resistance are believed to be the basic factors for the development of met-s [2]. Globally, the adult population met-s prevalence ranges from 20–25% [3].

The met-s was believed to be a challenge mostly faced by developed nations [4]. However, a few studies conducted have shown that met-s is increasing threateningly and is becoming more common in Africa, where it was considered rare. The rise in the incidence of met-s in Africa is believed to be due to a shift to modern ways of life, sedentary daily routines, and urbanization [5]. With the growth of the Western lifestyle came an increase in the consumption of food that is rich in calories but poor in fiber, as well as a decline in physical activity brought on by mechanized transportation and sedentary leisure activities [6]. Moreover, the demographic transition with the increasing number of elderly people is also playing its own role [4].

A systematic review and meta-analysis report on the prevalence of met-s in African populations demonstrated 32.4% of the overall prevalence, with Algeria having the greatest prevalence at 43.9% and Sudan having the lowest at 11.5%. The same report indicated a combined prevalence of 30.3% in East and Central Africa [7]. Moreover, according to projections of global mortality and burden of disease from 2002 to 2030, it is predicted that in sub-Saharan Africa, non-communicable diseases are expected to exceed communicable diseases by 2030 [8].

In Somaliland, according to the first ever held Health and Demographic Survey (HDS) in 2020, the prevalence of chronic NCDs was 7%, in which hypertension was the top ranking with 41% of all NCDs cases, followed by diabetes (19.1%), kidney diseases (9.1%), and heart diseases (6.9%) [9].

The level of met-s and related risk factors have not been studied in Hargeisa, Somaliland. Thus, there is a need to explore the prevalence and determinants with a view of suggesting measures for reversing met-s, changing lifestyles and risk factors related to it, and reducing adult premature deaths associated with NCDs. The research explores factors contributing to the growing public health challenge of met-s, providing evidence for improving general health care and promotion strategies in such places where the general health status of the adult population is low. It offers insights for policymakers and organizations in setting strategies and contributes to existing knowledge.

By considering this gap in the studies and given the significance in developing health promotion and chronic disease prevention programs in developing countries, we planned to conduct the study. Therefore, the current study aimed to assess the determinants and associated factors of met-s among the adult population in Hargeisa, Somaliland.

## Materials and methods

### Study design and period

A community-based cross-sectional study among the general adult population living in Hargeisa, Somaliland, was carried out from August 5^th^ to September 28^th^, 2023. Somaliland is a self-proclaimed de facto independent state in the Horn of Africa that broke away from Somalia in 1991. Somaliland has a population of 5.7 million as of 2021. It is a homogenous country dominated by Somali people. Islam is the official religion [10]. Hargeisa is the capital of Somaliland, with a population of 1.2 million. Hargeisa municipality is divided into eight districts, which contain various sub-districts [11].

### Study population

The study population was sampled from all adult populations living in Hargeisa for at least 6 months who were able and willing to respond. Those who were pregnant, too sick to respond, or had incomplete laboratory tests were excluded from the study.

The sample size was calculated using a single population proportion formula by considering the prevalence of met-s (17.9%) [12], according to International Diabetic Federation (IDF) criteria. Assuming a 95% confidence level, a margin of error of 5%, a 10% nonresponse rate, and a design effect of 2, the calculated sample size was 498.

$$n=\frac{{\left(Z_{\alpha /2}\right)}^{2}p(1‐p)}{d^{2}}=\frac{{\left(1.96\right)}^{2}*0.179(1‐0.179)\approx 226}{{\left(0.05\right)}^{2}}$$

226+22.6(10%non‐response)≈249

$$249*2\;(design\;effect)=\underline{\underline{498}}$$

**Fig 1:**
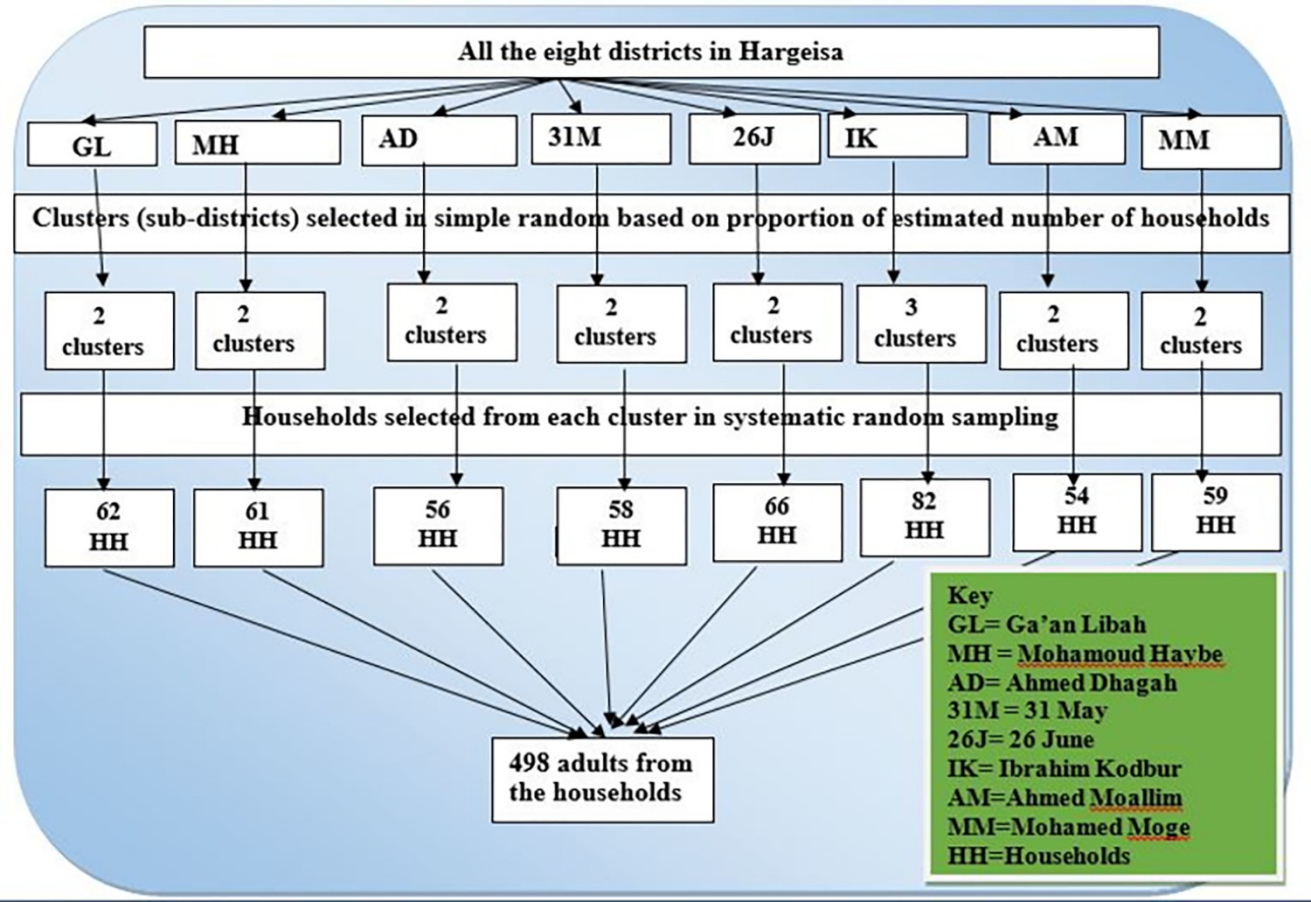
Schematic presentation of sampling procedure for the selection of study units, Hargeisa, Somaliland, 2023.

A multistage sampling scheme was employed, involving various sub-districts/clusters across all eight districts of the municipality. First of all, a total of 17 clusters in the eight districts were selected proportionally, using simple random sampling technique, based on the estimated number of households in each district; hence, 2 clusters were selected from each of Ga’an Libah, Mohamoud Haybe, Ahmed Dhagah, 31 May, Mohamed Moge, Ahmed Moallim, and 26 June district districts; and 3 clusters from Ibrahim Kodbur. In order to choose the households from the selected clusters, in the second stage, a systematic random selection technique was used within the respective clusters. In the selected households, where there were more than one eligible adults, we used the lottery method to randomly pick one adult person (Fig 1).

### Data collection and variables

A structured questionnaire was used to collect data in accordance with the STEPwise approach of the World Health Organization (WHO) for NCD surveillance in developing countries [13]. The approach has three levels. The first is a questionnaire to collect demographic, dietary, lifestyle, and behavioral information. The second was simple physical measurements, and the third was biochemical (laboratory) tests. Questions about substance use, such as khat use (a stimulant plant widely used in East African countries), were incorporated to adjust to local settings.

Participants were interviewed by experienced and trained health professionals. The questionnaire was initially prepared in English and then translated to Somali and retranslated back to English. The data were collected and supervised by health professionals. A 2-day training was done on the content of the questionnaire, data collection techniques, and the ethical conduct of human research. Before the actual data collection, the questionnaire and all data collection instruments were pretested outside the study area in a nearby town. A few amendments were made after pretesting.

Trained nurses and laboratory technologists performed physical/anthropometric assessments and blood sample collection. Blood pressure was measured in the sitting position by a digital BP apparatus after at least 5 minutes of rest. Three measurements were taken, and the mean value was recorded. Weight was assessed using a digital measuring scale with the individuals barefooted and wearing light clothing. Height was also measured using a calibrated instrument with regular procedures. The midpoint between the top of the iliac crest (hip bone) and the lower border of the last palpable rib was used to measure waist circumference. Body mass index (BMI) was calculated and grouped into underweight <18.5, normal 18.5–24.9, overweight ≥25, and obesity ≥30, according to WHO criteria. The Sedentary Behavior Questionnaire was used to assess sedentary behavior. It was measured on weekdays and weekends separately and analysed with weighted values to obtain accurate values [14].

After at least 8 hours of overnight fasting, a 10 ml blood sample was taken from each participant in their own household with proper materials and procedures to test serum triglycerides (TGs), high-density lipoprotein cholesterol (HDL-C), and fasting serum glucose (FG). Then the collected sample was sent to the laboratory, and tests were done using standardized medical laboratory instruments, procedures, and proper guidelines. Agape Mispaviva clinical chemistry analyser, Switzerland, was used to test TGs and HDL-C. For all areas of measurement and quality assurance, standard operating procedures were strictly followed.

There are several definitions of metabolic syndrome out there, and we mainly used the IDF criteria. It incorporates various ethnicities of different geographic regions of the world for the definition of abdominal obesity. In accordance with the IDF criteria, subjects were classified as having met-s if they had abdominal obesity (defined as waist circumference of ≥94 cm for men and ≥80 cm for women) plus two of any of the following risk factors: raised TG level (≥150 mg/dL) or specific treatment for this lipid abnormality; reduced HDL-C (<40 mg/dL in males and <50 mg/dL in females) or specific treatment for this lipid abnormality; raised blood pressure (systolic BP ≥130 mmHg or diastolic BP ≥85 mmHg) or treatment of previously diagnosed hypertension; and raised FG (≥100 mg/dL) or previously diagnosed with type 2 diabetes [3]. Then, the data were analysed, grouped, and dichotomized into having or not having met-s.

Various criteria are used for the definition of metabolic syndrome, in which NCEP-ATPIII (National Cholesterol Education Program–Adult Treatment Panel) and IDF are widely used in numerous literatures. The IDF established abdominal obesity as a requirement for the diagnosis of metabolic syndrome, emphasizing the waist circumference measurement as a quick screening. While abdominal obesity was classified differently in the ATPIII and IDF, the remaining four components of metabolic syndrome remained of the same value. The ATPIII suggested cut-off points of 102 cm for men and 88 cm for waist circumference, but the IDF recommended 94 cm for men and 80 cm for women. Until more precise statistics become available, the IDF advised using European data (94 cm for men and 80 cm for women) for sub-Saharan Africans. According to ATPIII, three of the five components of metabolic syndrome are required, while in IDF, central obesity and two the other four elements are required [15].

### Data processing and analysis

After data collection, each questionnaire was checked for completeness, clarity, and consistency. Then, a code was given before data entry. The data were cleaned and explored for outliers and missed values before entry into the Epi-data statistical package. Then, the data were exported and analysed using SPSS version 25 statistical packages. Different frequency tables, graphs, and descriptive summaries were used to describe the study variables. Bivariate analysis was performed to determine the existence of associations between dependent and independent variables. Binary logistic regression was performed to assess the strength of the association between each major independent variable and the outcome variables. Then, those variables with a P-value of less than 0.2 were included in a single model, and multiple logistic regression was performed. Finally, only those independent variables that maintain their association with outcome variables were used to construct the final models in multiple regressions. Odds ratios with P values and confidence intervals were used in each logistic regression analysis. A P-value less than 0.05 was considered statistically significant.

### Ethical consideration

The study obtained ethical clearance from the Research Ethics Committee of the University of Hargeisa Research and Community Service Directorate (Ref: DRCS/199/08/2023). Permission was obtained from the Somaliland Ministry of Health Development and all concerned bodies after discussion of the purpose of the study. For all participants, the aim of the study was explained, and they were reassured that their responses and results would be used only for research purposes and would remain confidential. Similarly, after a clear discussion about the purpose of the study, written consent was obtained from each study subject, and the subject’s right to refuse was respected. To assure the confidentiality of the study subjects’ responses, writing their names or any identification in the questionnaire was not needed. All ethical research protocols were maintained according to the Helsinki Declaration and the ethical rules of the university.

## Result

### Sociodemographic characteristics

In total, 498 adults participated in the study, making the response rate 100%. The mean and median of their ages were 42 and 39 years, respectively, with a standard deviation of 14. The age of the study participants ranged from 19 to 82 years, with slightly more than half (51%) belonging to the 25–34 and 35–44 year groups. Most study participants were married (85.3%) and female (56.2%). Of the respondents, 26.9% had secondary education, while 57.4% reported a monthly family income of 100–500 US dollars. All participants were Muslims by religion (Table 1).

**Table 1:** Sociodemographic characteristics of respondents, Hargeisa town, 2023.

Variables	Category	Frequency	%
Age	19–24	42	8.4
	25–34	131	26.3
	35–44	123	24.7
	45–54	91	18.3
	55–64	73	14.7
	>64	38	7.6
Marital status	Never married	52	10.2
	Married	424	85.3
	Divorced/widowed	22	4.4
Sex	Male	218	43.8
	Female	280	56.2
Educational status	No school	85	17.1
	Primary school	108	21.7
	Secondary school	134	26.9
	University	171	34.3
Average monthly income in US dollars	100–500	286	57.4
	501–1000	183	36.7
	>1000	29	5.8

### Lifestyle and behavioral characteristics

Among our study participants, 17.3% were khat users, while 12.4% were smokers. Use of khat and smoking were lower among women, at 1.8% and 1.2% of all respondents, respectively. None of our study participants reported drinking alcohol. Self-reported excessive salt use was 18.9%. Out of all the study subjects, 81.2% reported consumption of an average of less than 5 servings of fruits per day, and 80.9% reported less than 5 servings of vegetables. Only 61 respondents (12.2%) reported that their work involved vigorous or moderate physical activities. On the other hand, 92 participants (18.4%) reported doing vigorous or moderate regular physical exercise, of whom 7.6% of all respondents were females and 10.8% were males. On the other hand, as few as 7.2% reported that they used walking for transportation, and 10.4% reported that they spent 8 or more hours sitting sedentarily (Table 2).

**Table 2:** Lifestyle and behavioral characteristics of the respondents, Hargeisa, 2023.

Variables	Category	Frequency	%
Khat Use	Yes	86	17.3
	No	412	82.7
Smoking	Yes	62	12.4
	No	436	87.6
Physical activity at work	Low/none	437	87.8
	Moderate	47	9.4
	Vigorous	14	2.8
Physical exercise	Low/none	406	81.6
	Moderate	62	12.4
	Vigorous	30	6
Average time spent sitting in a day	<8 hours per day	446	89.6
	≥8 hours per day	52	10.4
Average servings of fruit per day	Less than 3 servings	216	43.4
	3–4 servings	188	37.8
	5 or more servings	94	18.8
Number of days fruits are eaten	<3 days per week	226	45.4
	3–4 days per week	194	39
	5 or more days per week	78	15.6
Average servings of vegetables per day	Less than 3 servings	265	53.2
	3–4 servings	138	27.7
	5 or more servings	95	19.1
Number of days vegetables are eaten	<3 days per week	195	39.2
	3–4 days per week	212	42.6
	5 or more days per week	91	18.3
Self-reported salt use	Far too much	5	1
	Too much	89	17.9
	Just the right amount	267	53.6
	Too little	132	26.5
	Far too little	5	1
Walking for transportation	Yes	36	7.2
	No	462	92.8

### Components of metabolic syndrome and its prevalence

**Fig 2:**
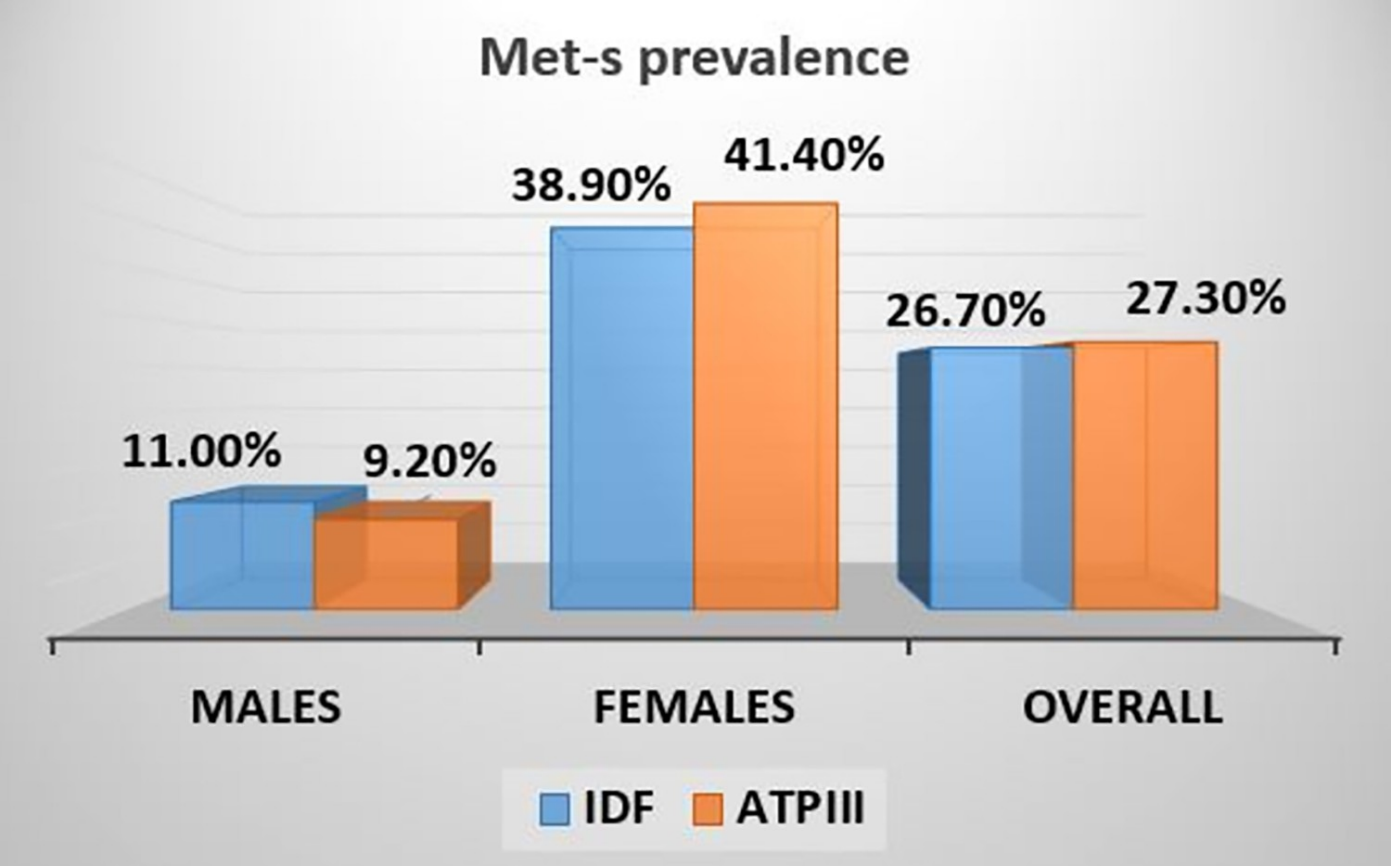
Prevalence of metabolic syndrome, Hargeisa town, 2023.

The overall prevalence of metabolic syndrome was calculated to be 26.7% using IDF criteria, 11% among males, and 38.9% among females. And, according to the ATPIII criteria, the prevalence was 27.3% overall, with males at 9.2% and females at 41.4% (Fig 2).

**Fig 3:**
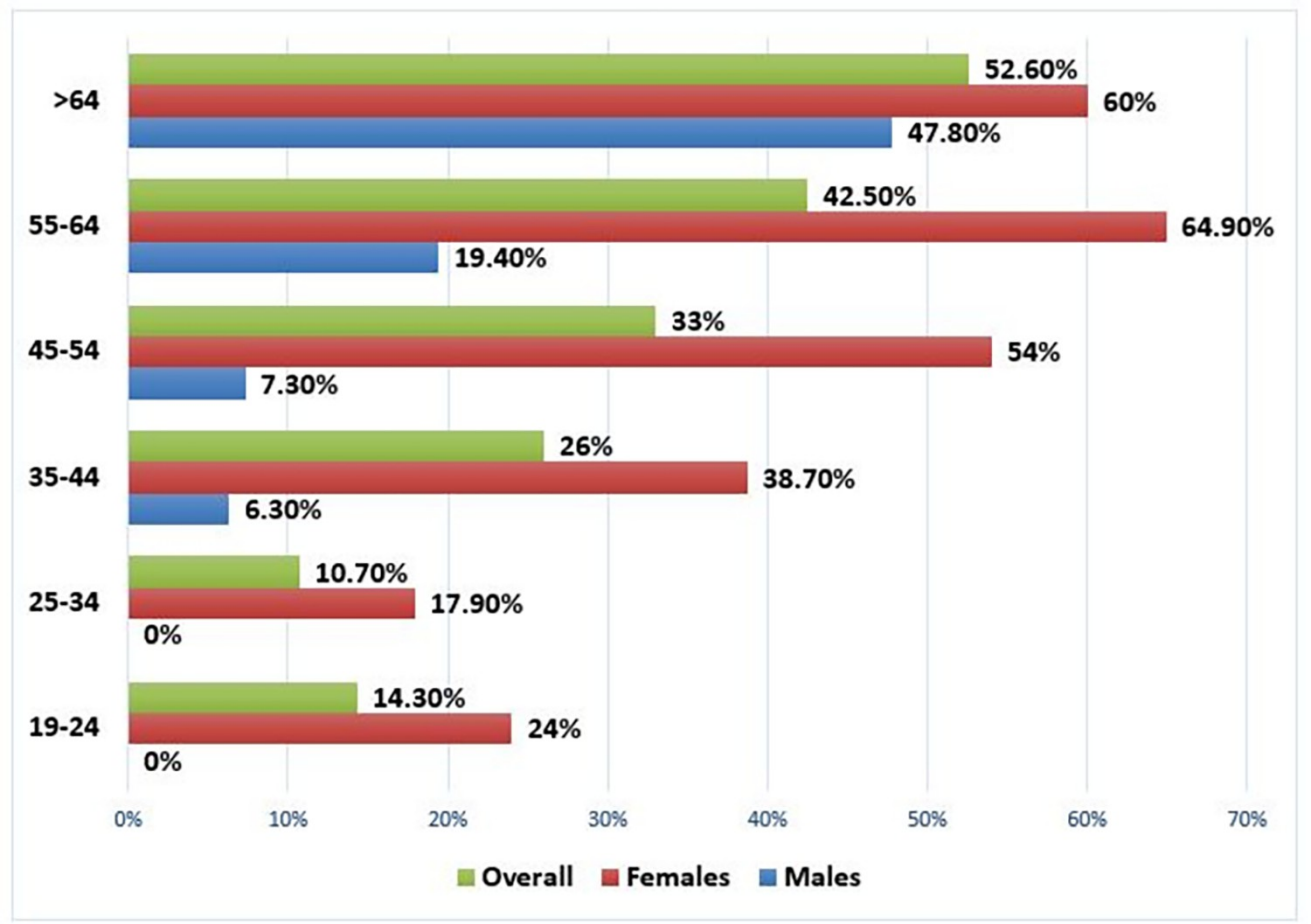
Gender difference in age-adjusted prevalence of met-s, Hargeisa, 2023.

The prevalence of metabolic syndrome increased with increasing age; hence, the highest prevalence of 52.6% was observed among people aged more than 64, followed by 42.5% among those aged 55–64 years. Regarding men, the highest rate of met-s was detected among those above 64 years old, while in women, it was 55–64 years old (Fig 3).

Among the five components of metabolic syndrome, abdominal obesity was found to be the top-ranked, accounting for 45.2% of all respondents using IDF criteria, followed by increased blood pressure (44.4%), high fasting blood glucose (41.4%), an abnormally low level of HDL (37.3%), and a higher level of triglycerides (10.8%). Abdominal obesity ranked the highest in females, and increased blood glucose was the highest in males. The overall prevalence of abdominal obesity differed between IDF and ATPIII criteria due to the different cut-points they employ (Table 3).

**Table 3:** Gender variation in met-s components, Hargeisa, 2023.

Met-S components	Males	Females	Overall
Abdominal obesity	18.8% (IDF) 10.6% (ATP)	65.7% (IDF) 58.9% (ATPIII)	45.2% (IDF) 37.8% (ATPIII)
Increased BP	31.7%	54.3%	44.4%
High FBS	29.4%	50.7%	41.4%
Low HDL	27.1%	45.6%	37.3%
Increased Triglycerides	11%	10.7%	10.8%

In 21.9% of participants, no component of metabolic syndrome was identified, while in 4.4%, all five components were detected. Nearly half of the respondents (48%) had 1 to 2 components of metabolic syndrome. In most cases, females appeared to lead males in the prevalence of each component (Table 4).

**Table 4:** Gender variation in the number of met-s components, Hargeisa, 2023.

Number of met-s components	Males	Females	Overall
0	8.6%	21.9%	39%
1	23.2%	24.5%	26.1%
2	25.4%	23.5%	21.1%
3	24.3%	17.3%	8.3%
4	12.9%	8.4%	2.8%
5	5.7%	4.4%	2.8%

Although the mean age of the men (43.8 years) was slightly greater than that of the women (40.8 years), more women had abnormal mean values in most components of met-s compared to men, leading to a higher prevalence of met-s in women. For instance, the mean BMI was 26.4 for women and 24.2 for men, while the overall BMI was 25.5. Similarly, the mean waist circumference was 90.4 cm for women and 87 cm for men (Table 5).

**Table 5:** Gender variation in mean values of met-s components, Hargeisa, 2023.

Variable	Males	Females	Overall
Age	43.8	40.8	42.1
BMI	24.2	26.4	25.5
Systolic BP	120	131	126.7
Diastolic BP	77	80	79
Waist circumference	87	90.4	89
Triglycerides	114	101	107
HDL	49	47	48
FBS	102	117	111

**Fig 4:**
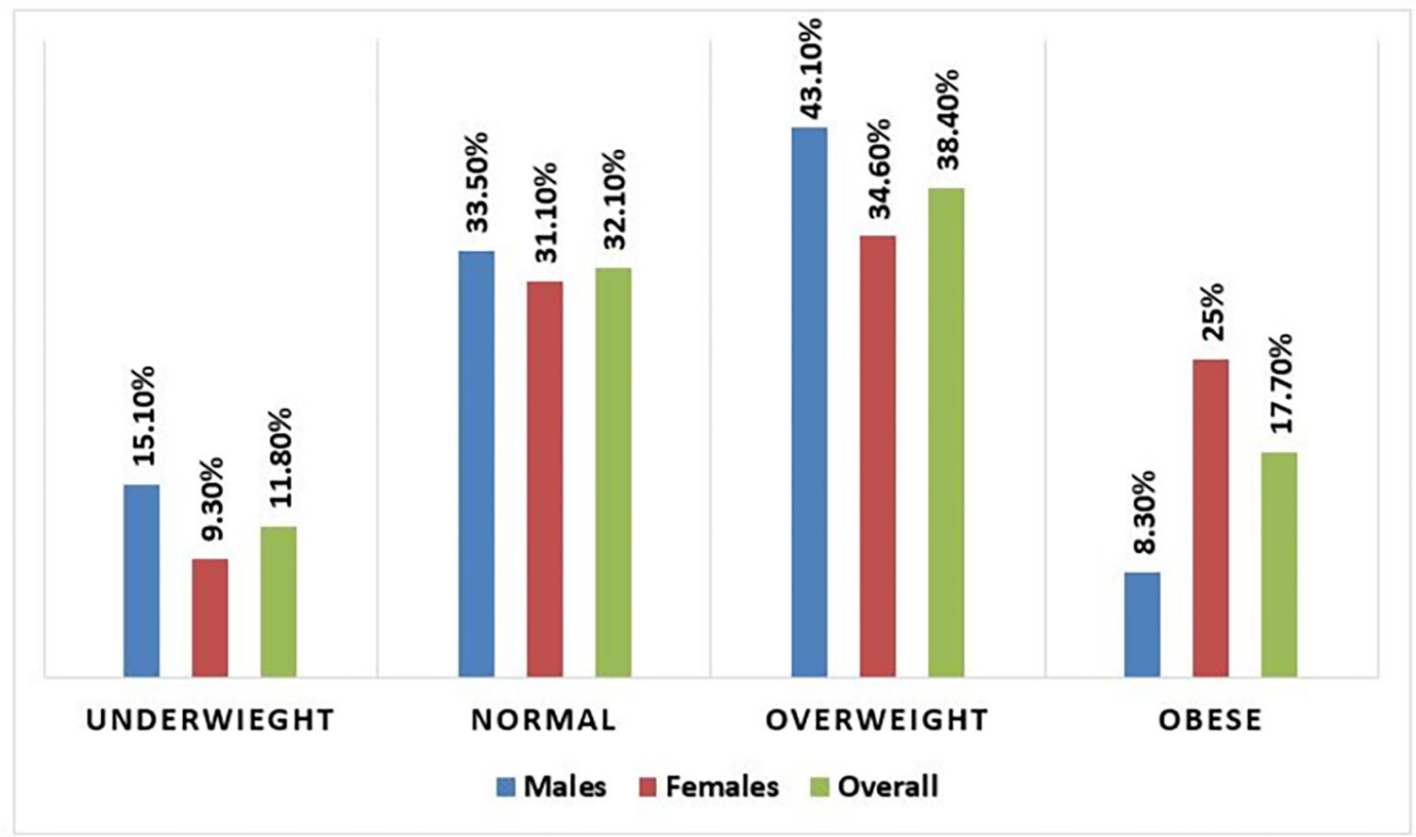
Gender variation in BMI value, Hargeisa, 2023.

Concerning BMI, 33.5% of men and 31.1% of women had nearly equal percentages of normal values (18.5–24.9). Twenty-five percent of women were obese, compared to only 8.3% of men (Fig 4).

### Factors associated with met-s

Bivariate and multivariate analyses were performed using logistic regression to analyse factors associated with met-s and rule out possible confounders. In the bivariate analysis, age, sex, educational status, obesity, sedentary behaviour, and lack of physical exercise were associated with the Met-S. However, the multivariate analysis revealed that becoming older, being a woman, being overweight or obese, sedentary behaviour, and a lack of physical exercise were associated with met-s.

Concerning gender, women (AOR = 10.8, CI 5.3–21.8, P < 0.001) were 10.8 times more likely to have met-s than men. Older people aged 45–54 years (AOR = 3.6, CI 1.17–11.27, P = 0.025), 55–64 years (AOR = 6.1, CI 1.98–19.8, P = 0.003), and older than 64 years (AOR = 9.1, CI 2.41–34.92, P = 0.00) were more likely to have met-s relative to their younger counterparts of 19–24 years.

Participants with a BMI of 25 or more (overweight or obese) (AOR = 4.5, CI 2.5–8, P < 0.001) were 4.5 times more likely to get met-s relative to people with a BMI of less than 25. Respondents who sat sedentarily for an average of 8 hours or more daily (AOR = 3.5, CI 1.6–7.5, P = 0.001) were 3.5 times more likely to get met-s than those who sat less than 8 hours. Study subjects who performed vigorous or moderate-intensity exercise (AOR = 0.39, CI 0.17–0.88, P = 0.024) were less likely to have met-s than those who did not perform it (Table 6).

**Table 6:** Bivariate and multivariate analysis showing factors associated with met-s, Hargeisa town, Somaliland, 2023.

Variables	Status of met-s		Crude Odds Ratio (COR) (95% CI) P Value	Adjusted Odds Ratio (AOR) (95% CI) P Value
	No met-s (%)	Met-s (%)		
**Gender**				
Male	194 (89%)	24 (11%)	1	1
Female	171 (61.1%)	109 (38.9%)	5.1 (3.16–8.38) 0.00*	10.8 (5.3–21.8) 0.00*
**Age**				
19–24	36 (85.7%)	6 (14.3%)	1	1
25–34	117 (89.3%)	14 (10.7%)	0.7 (0.25–2) 0.52	0.78 (0.24–2.48) 0.67
35–44	91 (74%)	32 (26%)	2.1 (0.81–5.4) 0.12	2.3 (0.73–6.97) 0.128
45–54	61 (67%)	30 (33%)	2.9 (1.1–7.7) 0.029*	3.6 (1.17–11.27) 0.025*
55–64	42 (57.5%)	31 (42.5%)	4.4 (1.6–11.8) 0.003*	6.1 (1.88–19.83) 0.003*
>64	18 (47.4%)	20 (52.6%)	6.6 (2.2–19.5) 0.001*	9.1 (2.41–34.92) 0.00*
**Educational status**				
No School	56 (65.9%)	29 (34.1%)	3 (1.6–5.6) 0.00*	
Primary school	74 (68.5%)	34 (31.5%)	2.6 (1.5–4.8) 0.001*	
Secondary	89 (66.4%)	45 (33.6%)	2.9 (1.7–5.1) 0.00*	
University	146 (85.4%)	25 (14.6%)	1	
**BMI**				
<25	196 (89.5%)	23 (10.5%)	1	1
> = 25	169 (60.6%)	110 (39.4%)	5.5 (3.4–9) 0.00*	4.5 (2.5–8) 0.00*
**Sedentary behavior**				
Less than 8 hrs	346 (77.6%)	100 (22.4%)	1	1
8 or more	19 (36.5%)	33 (63.5%)	6 (3.3–11) 0.00*	3.5 (1.6–7.5) 0.001*
**Physical exercise**				
Vigorous / moderate	82 (89.1%)	10 (10.9%)	1	1
Low or none	283 (69.7%)	123 (30.3%)	3.5 (1.78–7.1)0.00*	2.5 (1.1–5.7) 0.024*

*Statistically significant (P<0.05).

## Discussion

The study was aimed at assessing the determinants and prevalence of met-s among the general adult population in Hargeisa town, the capital of Somaliland, in 2023. Our results established that females are at higher risk for met-s. Moreover, the study indicated that people with advanced age, overweight and obesity, and more than 8 hours of sedentary behavior were at higher risk for met-s. In contrast, participants with active physical exercise were found to be at lower risk for met-s.

Globally, metabolic syndrome is very common. Depending on the diagnostic criteria, a meta-analysis of world-wide data from 28 million persons in the general population revealed a prevalence ranging from 12.5% to 31.0% [16]. It is extremely worrisome that a lot of people are being impacted by this condition. Metabolic syndrome is no longer considered a problem of a certain geographic region or ethnicity. Metabolic syndrome is a world-wide public health challenge that affects people of all ages and is present in both developing and developed nations.

In our study, the total prevalence of metabolic syndrome was 26.7% using IDF criteria, 11% among males, and 38.9% among females. The ATPIII criteria yielded a total prevalence of 27.3%, which is not far from the IDF criteria prevalence. Between the two genders, there was a difference in the gender specific prevalence of met-s. Male prevalence decreased to 9.2% in ATPIII criteria from 11% of the IDF, while female prevalence rose to 41.4% in ATPIII from 38.9% of the IDF. The disparities in the overall and gender-specific prevalence of metabolic syndrome are not surprising, as there are different criteria and cut-points used by IDF and ATPIII. The IDF criteria necessitates central obesity in order to determine the occurrence of metabolic syndrome, which accounts for these discrepancies in the results, as the participants will not be classified as having metabolic syndrome if central obesity is not present, even if the values of the other components are higher than the normal.

Our finding that showed a 26.7% prevalence rate of met-s is comparable with a community-based cross-sectional study performed in Kenya [17], in which the prevalence of met-s was 25.6%. Similarly, our finding is not too far from the figures in a hospital-based study conducted in Addis Ababa with a met-s of 20.3% [18], and among Haramaya University workers in Ethiopia with a prevalence of 20.1% [4]. Our finding is much lower compared to a report in Dessie [19], which indicated a prevalence of 59.4%. This vast disparity could be related to variations in sampling methods and diagnostic criteria. The study in Dessie employed ATPIII criteria, and the sample population comprised hospital-based type 2 diabetic patients; hence, a higher met-s rate is anticipated. In contrast, our study’s finding is higher than the 17.9% seen in Addis Ababa working adults [12]. The difference could be attributed to differences in the study settings, sociocultural context, and lifestyles.

People with advanced age had a higher rate of met-s, and we confirmed a steady increase across age groups in the met-s rate, particularly among people older than 45 years. Comparable findings were reported in various community-based studies conducted in India [20] and Bangladesh [21], and a facility-based study in Addis Ababa [18]. Being elderly is associated with degeneration of body tissues that might adversely affect health over time and through the natural process of ageing.

Obese participants had higher odds of met-s according to our investigation. This is in line with a finding from Dessie [19], which indicated the same. Similar findings were also reported in community-based studies conducted in Pakistan [22] and Addis Ababa [12]. Although obesity is a modifiable risk factor of met-s, many people continue to live with it, increasing their likelihoods of developing chronic non-communicable diseases.

The American Heart Association (AHA) recommends 4 servings of fruits and 5 servings of vegetables per day based on the 2000 calorie/day eating pattern [23]. However, in our study, we found out that nearly half (43.4%) and more than half (53%) of the respondents reported that they consume less than 3 servings of fruits and vegetables per day, respectively, which is too far behind the recommended amount. In addition to that, the study participants who consumed fruit and vegetables five or more days per week were only 15.6% and 18.3%, respectively. That means a huge majority of the study participants were not consuming an adequate amount of fruits and vegetables as per the standard. Such below-the-recommended standard consumption of fruits and vegetables was also reported in studies such as those in Addis Ababa [18], in which only 1.5% reported eating 5 or more servings of fruits per day. Another study in Haramaya [4] also found only 8% reported eating 5 or more servings of fruits and vegetables per day. Fruits and vegetables include essential vitamins, minerals, and plant compounds. They also contain fiber. It has long been known that a fruit and vegetable rich diet can help avoid a variety of non-communicable diseases.

In this study, the overall prevalence of met-s was 26.7%. Men make up just 11%, with women accounting for the majority at 38.9%. This indicates met-s has a greater tendency to affect women than men. Community-based research conducted in Brazil [24], India [20], Kenya [19], workers at Haramaya University [4], and working adults [12] revealed similar findings of more women impacted by met-s. Men were shown to be less likely to be obese and more likely to be physically active than women in several research studies, including our own. This could be one of the reasons for differences in the level of met-s between the genders.

According to the WHO, physical inactivity raises the risk of NCDs and other detrimental health effects, while physical activity is helpful to health and well-being. Sedentary lifestyles and physical inactivity combined are making NCDs more common and burdening healthcare systems [25]. Physical inactivity was found to be one of the factors significantly associated with a greater risk of the development of met-s in our study.

Sedentary lifestyle favoured the development of chronic NCDs and met-s, particularly at middle or later age, as our and other investigations have shown; hence, more than 8 hours of passive, sedentary sitting was associated with increased odds of met-s. This is consistent with a study done among Haramaya University employees [4].

### Limitations and strength of the study

#### Limitations

The study’s cross-sectional design might make it difficult to determine a causal relationship between the independent variables and dependent. Furthermore, the source of some information was based merely on respondents’ self-reported data. There might be social desirability and recall biases as well.

#### Strength

We employed a community-based study in the general adult population, in order to enhance the generalizability of the findings and offer a comprehensive picture of the problem within the community. Variations between the two genders about the prevalence and each of the five elements were examined and presented.

Considering the aforementioned limitations and strengths, we conclude that this study addressed a major gap in the current understanding of met-s and related risk factors, and has significance for local and regional metabolic syndrome prevention initiatives.

## Conclusion

Met-S was found to be predominant, affecting more than a quarter of the general adult population in Hargeisa: 26.7% using IDF criteria and 27.3% using ATPIII. Met-s was significantly associated with being a woman, advanced age, sedentary behavior, physical inactivity, and obesity. There was marked gender gap in prevalence of met-s, with females being more affected. The study emphasizes the need for community-based prevention strategies and health promotion activities to reduce met-s. It stresses the need for public education, policy design, behavioural change interventions, screening, and targeting susceptible populations. It recommends conducting related studies among different population segments and in regions across the Somaliland.

## Supporting information

S1 File SPSS dataset-BPCR. (SAV)

S2 File Questionnaire MetS final. (DOCX)
