# Supplementary material for: Determinants and prevalence of metabolic syndrome among the adult population in Hargeisa, Somaliland: A community-based cross-sectional study
Source: PLoS One. 2024 Dec 27;19(12):e0316094. doi: 10.1371/journal.pone.0316094 (PMC11676556; doi:10.1371/journal.pone.0316094)
Supplement: S2 File — (DOCX) [file pone.0316094.s002.docx]

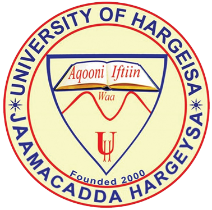


My name is ______________. I am working in research team of UoH Faculty of Health Sciences. I am here to study about **“**Determinants and Prevalence of Metabolic Syndrome among the Adult Population in Hargeisa, Somaliland” and you are chosen to participate in this study. You are selected and included in the study as part of the sample population to complete the questionnaire designed by the researcher.

The information obtained in this study will be used only for research purposes. Confidentiality is strictly protected and none of your response will be reported separately. Therefore, there is no need to tell or write your names or House ID numbers. It is your right to participate or to refuse in this study. And you can drop any individual question or the whole. But your participation in the study is very important to come up with important findings which may help health planners to intervene the problem.

So, please take a few minutes to answer, and your kind response and cooperation is highly appreciated.
If you agree, some questions related to lifestyles will be asked. Simple physical measurements such as height, weight, abdominal and hip circumference will be done. Your B/P will be measured. Your blood sample will be taken for laboratory test of FBS, and lipid profile. This would take about 15-20 minutes. There would be no hurt to you and your body. However, when blood sample is taken by needle inserted into your veins, you may feel some mild pain.

- Are you willing to participate in the study? Yes_______ No__________
- Signature of the interviewer certifying that informed consent has been given by respondent. ____
- Name of the interviewer _______________________ Sign__________ Date________
- Questionnaire ID _________ District name ________ Time started _______ Time finished ____
- Name of the supervisor _____________________________ Sign_____________ Date_______

**Part I: - Socio demographic Characteristics**

| **No** | **Questions** | **Response** | **Skip to** |
| --- | --- | --- | --- |
| Q101 | **Age** | **_______years** |  |
| Q102 | **Sex** | 1. **Male** 2. **Female** |  |
| Q103 | **Marital Status** | 1. **Never married** 2. **Currently married** 3. **Separated / Divorced** 4. **Widowed** |  |
| Q104 | **Your Educational status** | 1. **No schooling** 2. **Primary school** 3. **Secondary school** 4. **College / University** |  |
| Q105 | **Educational status of spouse (If relevant)** | 1. **No schooling** 2. **Primary school** 3. **Secondary school** 4. **College / University** |  |
| Q106 | **Number of people older than 18 years including you in household?** | **________________** |  |
| Q107 | **Total family size** | **________________** |  |
| Q108 | **Occupation** | 1. **Government / NGO employee** 2. **Self employed** 3. **Student** 4. **Retired** 5. **Unemployed (able to work)** 6. **Unemployed (unable to work)** 7. **Others, specify_____________** |  |
| Q109 | **Average monthly income** | **_________________ US Dollars** |  |
| Q110 | **Is your spouse employed?** | 1. **Yes** 2. **No** |  |

**Part II: - Behavioral Measurements – Smoking and Alcohol**

| **No** | **Questions** | **Response** | **Skip to** |
| --- | --- | --- | --- |

| Q201 | **Do you currently smoke any tobacco, such as cigarettes, or shisha?** | 1. **Yes** 2. **No** | Q209 |
| --- | --- | --- | --- |
| Q202 | **Have you ever consumed any alcohol such as beer, wine, spirits?** | 1. **Yes** 2. **No** | Q214 |
| Q203 | **Do you currently use khat?** | 1. **Yes** 2. **No** |  |

**Diet**

| **No** | **Questions** | **Response** |  |
| --- | --- | --- | --- |
| Q204 | **In a typical week, on how many days do you eat fruits?** | **______________ days** |  |
| Q205 | **How many servings of fruit do you eat on one of those days?** | **____________ number of servings** |  |
| Q206 | **In a typical week, on how many days do you eat vegetables?** | **______________ days** |  |
| Q207 | **How many servings of vegetables do you eat on one of those days?** | **____________ number of servings** |  |
| Q208 | **How much salt or salty sauce do you think you consume?** | 1. **Far too much** 2. **Too much** 3. **Just the right amount** 4. **Too little** 5. **Far too little** |  |

**Physical activity**

| **No** | **Questions** | **Response** |  |
| --- | --- | --- | --- |
| Q209 | **Do you walk or use a bicycle *(pedal cycle)* to get to places?** | 1. **Yes** 2. **No** | Q233 |
| Q210 | **Do you do any vigorous-intensity sports?** | 1. **Yes** 2. **No** | Q236 |
| Q211 | **How much time you spend doing vigorous-intensity sports in a day?** | **_____ hours or _____ minutes** |  |
| Q212 | **Do you do moderate-intensity sports?** | 1. **Yes** 2. **No** | Q239 |
| Q213 | **How much time you spend doing moderate-intensity sports in a day?** | **_____ hours or _____ minutes** |  |
| Q214 | **How much time do you usually spend sitting or reclining in a day? (Use SBQ)** | **_____ hours** |  |

**Step 2 and 3: - Physical and Biochemical measurements**

| Q301 | **B/P? (Within 5 minutes interval)** | **1^st^ measurement __________________ 2^nd^ measurement __________________** |  |
| --- | --- | --- | --- |
| Q302 | ***Pulse Rate*? (Within 5 minutes interval)** | **1^st^ measurement __________________ 2^nd^ measurement __________________** |  |
| Q304 | **Did you take any drug that affect the blood glucose today?** | 1. **Yes** 2. **No** |  |
| Q305 | **Weight __________ kgs**  **Height __________ cms**  **Waist __________ cms**  **Hip __________ cms** | |  |
| Q306 | **FBS _______________**  **Triglycerides _____________**  **HDL _____________**  Signature of Lab technician _________________ | |  |
